# Peripheral Low Level Chronic LPS Injection as a Model of Neutrophil Activation in the Periphery and Brain in Mice

**DOI:** 10.21203/rs.3.rs-3443401/v1

**Published:** 2023-10-19

**Authors:** Michelle Aries, Makayla Cook, Tiffany Hensley-McBain

**Affiliations:** McLaughlin Research Institute; McLaughlin Research Institute; McLaughlin Research Institute

## Abstract

Lipopolysaccharide-induced (LPS) inflammation is used as model to understand the role of inflammation in brain diseases. However, no studies have assessed the ability of peripheral low-level chronic LPS to induce neutrophil activation in the brain. Subclinical levels of LPS were injected intraperitoneally into mice to investigate impacts on neutrophil frequency and activation. Neutrophil activation, as measured by CD11b expression, peaked in the periphery after 4 weeks of weekly injections. Neutrophil frequency and activation increased in the periphery 4–12 hours and 4–8 hours after the fourth and final injection, respectively. Increased levels of G-CSF, TNFa, IL-6, and CXCL2 were observed in the plasma along with increased neutrophil elastase, a marker of neutrophil extracellular traps, peaking 4 hours following the final injection. Neutrophils and neutrophil activation were increased in the brain of LPS injected mice when compared to saline-injected mice 4 hours and 4–8 hours after the final injection, respectively. These results indicate that subclinical levels of peripheral LPS induces neutrophil activation in the periphery and brain. This model of chronic low-level systemic inflammation could be used to understand how neutrophils may act as mediators of the periphery-brain axis of inflammation with age and/or in mouse models of neurodegenerative or neuroinflammatory disease.

## Introduction

Neuroinflammation and immune activation are widely accepted as a significant contributing factor in the progression and degeneration of patients with Alzheimer’s Disease (AD), Parkinson’s Disease (PD), Multiple Sclerosis (MS), Amyotrophic Lateral Sclerosis (ALS), Huntington’s Disease (HD), and other neurodegenerative diseases^1–3^. Moreover, neuroinflammation and immune activation are present before the onset of symptoms^4,5^. An increase in proinflammatory cytokines, such as tumor necrosis factor-alpha (TNFα), and interleukin-1b (IL-1b), and caustic molecules released during inflammation, such as nitric oxide (NO) and myeloperoxidase (MPO), have been associated with a worse prognosis across multiple neurodegenerative diseases^2,6^. Neuroinflammatory markers such as these, along with immune cells, including neutrophils and microglia, are potential therapeutic targets and potential markers for early detection for multiple neurodegenerative diseases.

Multiple studies have correlated the onset and progression of neurodegenerative diseases with proinflammatory factors and immune cell activation and accumulation^7–9^. Transcriptomics studies have demonstrated an increase in pro-inflammatory differentially expressed genes (DEGs) in neurodegeneration, and genes involved in neutrophil activation and adhesion have been identified in areas with blood brain barrier (BBB) disruption and associated with disease progression^7,9–11^. Neutrophils are of therapeutic interest because they are the most abundant circulating leukocyte and have been shown in both human and mouse models to be associated with a worse prognosis in neurodegenerative diseases^12–14^. Further, humanized AD mouse model studies have shown an increase in short-term spatial memory and a decrease in capillary blood flow stalling when targeting neutrophil adhesion or accumulation^14–16^.

Neutrophils are essential to fight invaders and clear debris^17^. Neutrophils have three main ways in which they contain pathogens, including phagocytosis, degranulation, and formation of neutrophil extracellular traps (NETs). While neutrophils are important in fighting infection and repairing tissues, they can also cause damage to tissues through their release of antimicrobial peptides, enzymes meant to degrade extracellular matrix, and reactive oxygen species^18–20^. Neuroinflammation plays a role in neurodegenerative diseases, including both increased inflammatory signaling and dysregulation of resident glial cells in the brain and the infiltration of leukocytes from the periphery to the central nervous system (CNS)^21,22^. Neutrophils are not commonly found in a healthy brain because of their exclusion by the blood brain barrier (BBB)^17,23^. However, the BBB is disrupted in events such as an ischemic stroke^23–25^) and traumatic brain injury^24,25^, in brain diseases such as Alzheimer’s Disease (AD)^18,26^ and Parkinson’s Disease (PD)^27^, and in viral^25,28^, fungal and parasite infection^17^. Although neutrophils have been documented in the brain in several neuroinflammatory conditions and diseases, such as stroke, multiple sclerosis, and AD, the role of peripheral inflammation in neutrophil infiltration and activation in the brain is unclear^15,18,26,29,30^. Administration of high levels of lipopolysaccharide (LPS) into the periphery as a model of sepsis results in neutrophil infiltration into the brain^31–33^. However, the level of inflammation that is induced in sepsis models is not representative of the chronic low-level inflammation that occurs with age and likely contributes to age-related neurological disease. To date, there are no models of chronic peripheral inflammation and neutrophil brain infiltration and activation. With this pilot study, we demonstrate that low-level chronic inflammatory stimuli (LPS) in the periphery induces neutrophil infiltration and activation in the brain. This model can be used in future studies to investigate the role of neutrophils in neuroinflammation and related brain disorders; how peripheral mediators and genetic risk factors for disease may alter neutrophil responses to these stimuli; and if neutrophils are mechanistic drivers of the subsequent neuroinflammation and neurodegeneration.

## Results

### Determining the Optimal Duration of Peripheral Neutrophil Activation with Chronic Low-Level LPS

LPS is a component of the outer membrane of gram-negative bacteria that binds to toll-like receptor 4 (TLR-4) on immune cells. It is commonly used to induce inflammation in the periphery and brain. However, studies differ in their dose, frequency, and route of administration^3^. Most studies have investigated acute exposure to LPS, administering LPS intraperitoneally (IP) daily for 5–7 days at doses of 0.25–1 mg/kg. Chronic exposure studies have demonstrated microglial and astrocyte activation and memory impairment with similar doses administered IP once or twice weekly for 4–6 weeks^34^. However, no studies investigating chronic LPS-induced neuroinflammation and potential cognitive consequences conducted to date have investigated the induction of neutrophil activation in the periphery or brain. As such, we sought to investigate 0.5 mg/kg of LPS once per week as a potential model of neutrophil inflammation in the brain. This LPS exposure represents the threshold of physiological changes in mice, and can be used to model low-level chronic inflammation, as may occur in persons with microbiome alterations or frequent GI or respiratory infections common in elderly individuals^35^. We examined neutrophil frequency and neutrophil activation, via expression of CD11b, in response to this low level chronic LPS. CD11b is important for neutrophil migration and adhesion and is known to be increased in a TLR-4 dependent manner with LPS stimulation of neutrophils^36^. Importantly, CD11b expression is increased in persons with AD and correlates with disease severity, making it a relevant marker of neutrophil activation in models of neuroinflammation and neurodegeneration^37^.

**Figure 1:**
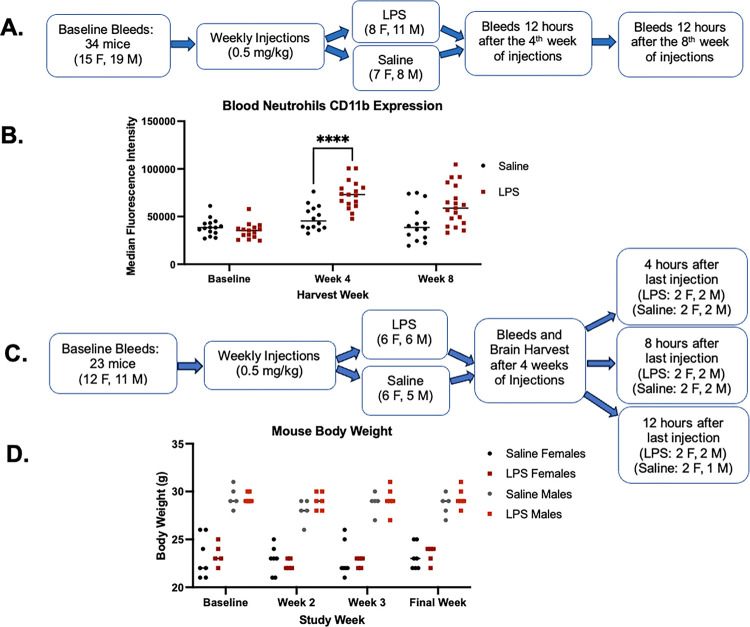
Study design iterations. A) Schematic overview of the preliminary LPS pilot study design to determine optimal number of injections. B) Median CD11b expression as measured by flow cytometry on peripheral neutrophils from the initial 8-week pilot described in (A). C) Schematic overview of study design for the next iteration of the study. Blood and brain tissue were collected after 4 weeks of injections. Neutrophil frequency, activation, and soluble markers were measured 4 hours, 8 hours, and 12 hours after the last injection. D) Body weight throughout the duration of study outlined in schematic C.

To determine the optimal duration of chronic LPS injections, male and female mice were IP injected with 0.5 mg/kg of LPS or saline once per week for 8 weeks, and blood was collected at baseline (pre-injection), 12 hours after the 4th injection, and 12 hours after the 8th injection (Fig. 1A). Following injections, neutrophil activation was assessed via flow cytometry. CD11b expression was highest on neutrophils after 4 weeks of injections, suggesting a potential for increased tolerance between 4 weeks and 8 weeks of injections (Fig. 1B). Based on these data, the next group of mice was injected for 4 weeks to investigate the timing of neutrophil activation in the brain following the final LPS injection (Fig. 1C). Blood was drawn at baseline and blood and brain tissue were collected 4 hours, 8 hours, or 12 hours after the 4th and final injection (Fig. 1C). As expected with this level of LPS, there were no significant changes in the body weight in either the saline- or LPS-injected mice throughout the experiment nor significant differences in body weight between the saline- and LPS-injected groups at any timepoint (Fig. 1D).

### Peripheral Neutrophil Activation Following Low-Level Chronic LPS

**Figure 2:**
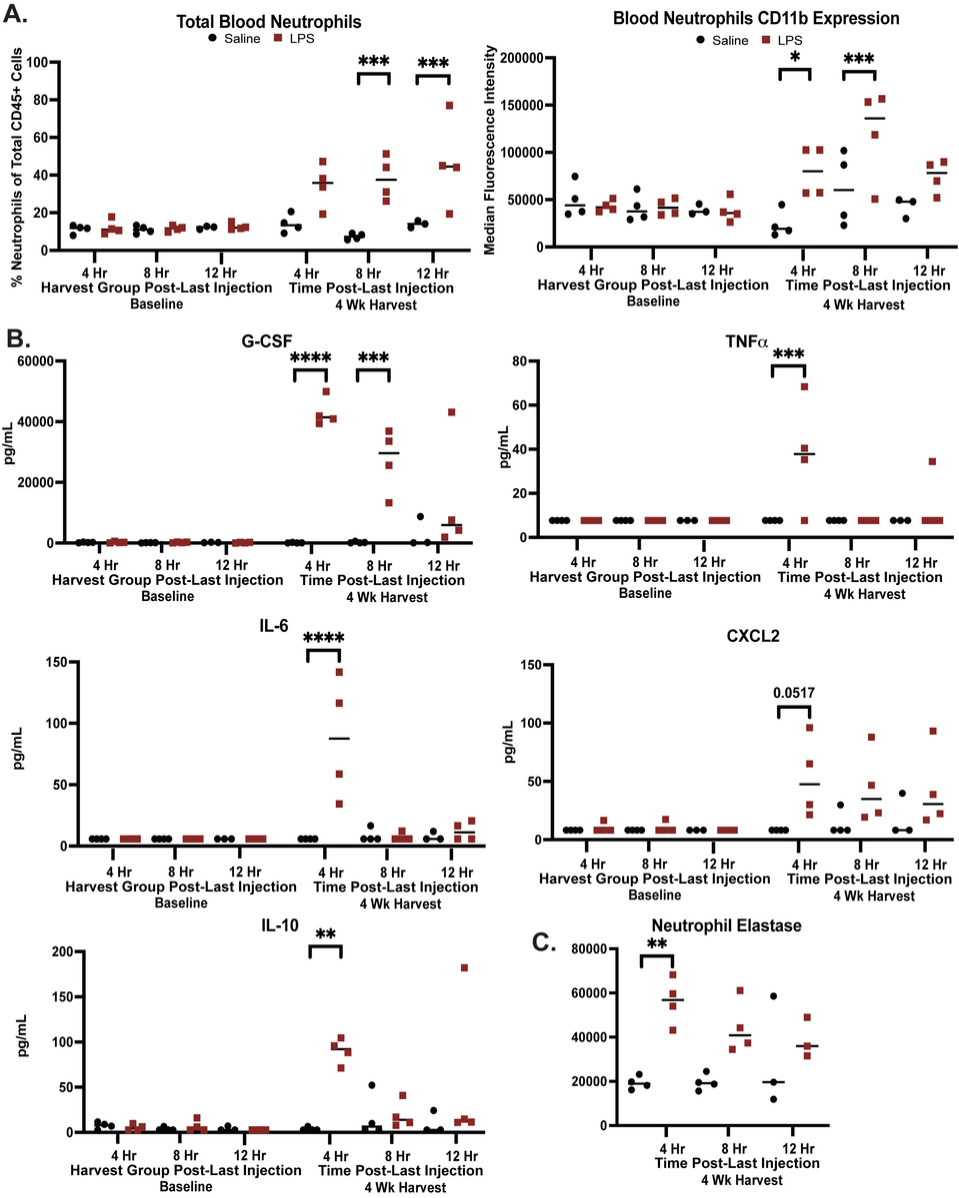
Increased peripheral neutrophil activation following 4 weeks of chronic low-level LPS injections. A) Neutrophil frequency in peripheral blood and median CD11b expression on peripheral blood neutrophils as measured by flow cytometry in mice receiving LPS (red) or saline (black). Neutrophils are defined as live, CD45+, CD11b+, Ly6G+ cells (see Supplementary Fig. 1). B) Increased cytokines and chemokines in plasma as measured by multiplex bead array (LegendPlex) in mice receiving LPS (red) or saline (black). C) Increased neutrophil elastase in plasma as measured by ELISA in mice receiving LPS (red) or saline (black). A and B were measured in submandibular blood and C was measured in additional cardiac blood collected at time of euthanasia. Statistical significance assessed by ANOVA followed by Tukey’s multiple comparisons test with multiplicity adjusted P values represented as * P < 0.05, ** P < 0.01, *** P < 0.001, **** p < 0.0001.

Following 4 weeks of IP LPS injections, blood neutrophil frequency and activation were assessed via flow cytometry (Supplementary Fig. 1). No increases in blood neutrophil frequency or activation were observed in the control mice receiving saline. Neutrophil levels increased 4 hours after the final LPS injection, with significant increases observed 8 and 12 hours following LPS injection (Fig. 2A). Similarly, neutrophil CD11b expression increased 4 hours after the final LPS injection, peaking 8 hours following LPS injection (Fig. 2A). Peripheral cytokines and chemokines with the ability to regulate neutrophil activation, maturation, and recruitment were measured in plasma via a LEGENDPlex to assess the potential role of soluble mediators of neutrophil activation. Of 12 total analytes measured, 5 showed a statistically significant change between the LPS and saline groups. G-CSF, TNFa, IL-6, and CXCL2, potential mediators of neutrophil activation, maturation, recruitment, increased 4 hours after LPS injection, with G-CSF remaining elevated zing by 8 hours post LPS administration (Fig. 2B). The anti-inflammatory mediator IL-10 was also increased 4 hours following the final LPS injection, suggesting a potential compensatory response early following LPS injection^4^. Finally, neutrophil elastase (NE), a marker of neutrophil extracellular trap release^5,6^, was measured via ELISA in plasma. NE was significantly increased 4 hours following LPS injection and remained elevated but lower 8 and 12-hours following post LPS 2C) frequency and neutrophil activation in the blood that peaked 8 hours after LPS administration. Soluble mediators of neutrophil responses and neutrophil activation begin to resolve 8–12 hours following LPS. However, neutrophil frequency remains elevated through 12 hours post-LPS, as neutrophils would not yet be anticipated to undergo homeostatic apoptosis by 12 hours following release into the periphery and apoptosis could be further delayed in response LPS^38^.

### Brain Neutrophil Activation Following Low-Level Chronic LPS

**Figure 3:**
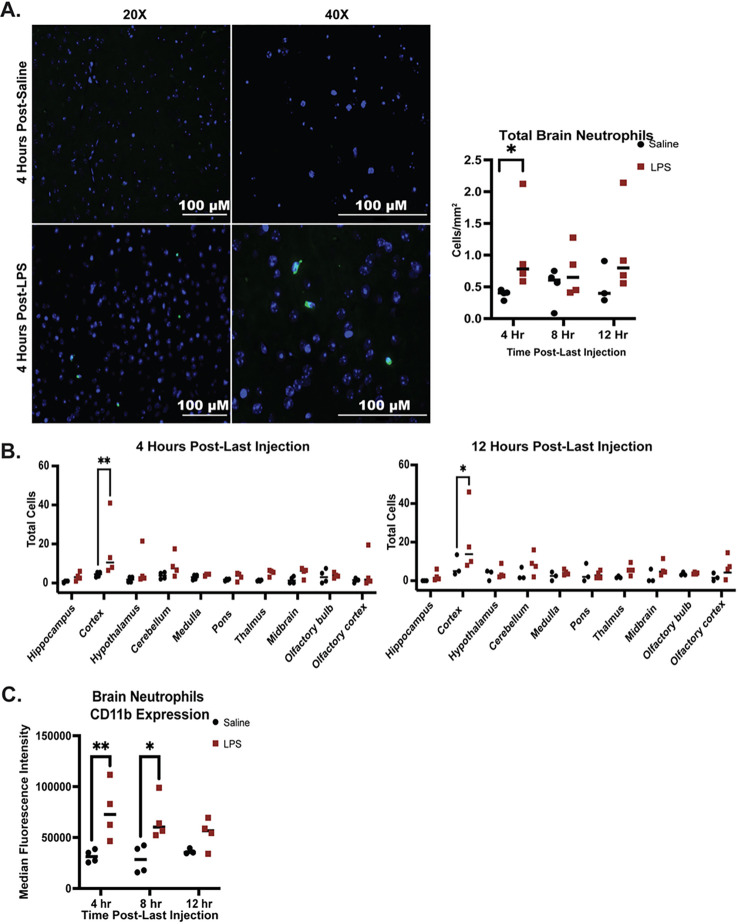
Infiltration of neutrophils into the brain and activation of brain neutrophils following 4 weeks of chronic low-level LPS injections. A) Left: Example images of neutrophils stained with MPO-specific antibody in the cortex of saline- and LPS-injected mice. Right: Neutrophils counted via microscopy in sagittal brain sections and normalized by area in saline- and LPS-injected mice. B) Neutrophils counted in each brain region 4 and 12 hours following the final injection. C) Neutrophils in the brain were assessed by flow cytometry (see Supplementary Fig. 1). Neutrophils were defined as CD45+CD11b+Ly6G+ cells and activation was assessed via CD11b expression.

Brain neutrophils were assessed after 4 weeks of LPS injections at 4 hours, 8 hours, and 12 hours following the final injection. Brain neutrophils were identified in sagittal sections via myeloperoxidase (MPO) staining using an antibody that has been validated to stain for neutrophils in mouse and human brain tissue (Fig. 3A)^18^. Neutrophils were counted across entire sagittal sections and were significantly elevated in the brain 4 hours following the final LPS injection. Neutrophil frequencies were higher but not significantly higher than saline-injected mice at 4 hours and 8 hours following LPS administration, as neutrophils were higher in the 8-hour and 12-hour groups of saline-injected mice. Of note, increased peripheral neutrophil activation in the 8-hour and 12-hour groups of saline-injected mice were also observed at time of collection, suggesting a potential for neutrophil activation and brain infiltration with handling and injection-induced inflammation (Fig. 2A). Increased concentrations of neutrophils in the brain were observed mainly in the cortex (Fig. 3B), which remained significantly elevated in LPS-injected mice compared to saline-injected mice through 12 hours post LPS (Fig. 3B). Neutrophil CD11b expression measured on neutrophils isolated from brain tissue was significantly elevated 4 hours and 8 hours following the final LPS injection, as was observed in blood neutrophils (Fig. 3C). Taken together these data indicate that neutrophils infiltrate the cortex and demonstrate increased activation in brain tissue by 4 hours following peripheral low-level LPS injection.

## Discussion

Despite LPS as a common model for peripheral immune activation and subsequent neuroinflammation, no studies have investigated the role of neutrophils in mediating the periphery-brain axis of inflammation induced by chronic low levels of LPS. Neutrophils in the periphery and vasculature could contribute to neuroinflammation and neurodegeneration in multiple ways, including their role in blood flow stalling and their direct release of proinflammatory cytokines and factors that disrupt the blood brain barrier^12,39^. In addition, peripheral inflammation could result in increased neutrophil adhesion molecules that result in their extravasation into the brain and release of granule and NET components that may directly damage tissue. Neutrophils in the brain could impact the surrounding tissue in multiple ways. Their release of ROS, NETs, and cytokines can activate microglia, and their release of extracellular histones can induce neuronal apoptosis ^17, 40^. The matrix metalloproteinases (MMPs) and proteases released by neutrophils during degranulation or the generation of NETs results in breakdown of the extracellular matrix and neuronal damage^17,23^.

With this study, we sought to determine if low levels of peripheral LPS results in neutrophilic contributions to neuroinflammation. LPS is often used a general inducer of chronic inflammation in animal models, and LPS, derived from resident gram-negative bacteria in the gut, mouth, and skin, is also specifically implicated in the pathogenesis of neurogenerative and neuroinflammatory diseases^41^. Models of various neurodegenerative diseases have demonstrated that chronic low-level LPS administered IP results in neuroinflammation, as evidenced by induced glial activation, cognitive dysfunction, cerebrovascular leakiness, and inflammatory cytokines in the brain^34^. Chronic peripheral LPS in these models also induced proteinopathy, with increased amyloid deposition in AD models and TDP-43 aggregation in ALS models^34^. The role of neutrophils in mediating CNS inflammation and neurodegeneration in response to low levels of chronic LPS in the periphery has never been examined. Here, we report that chronic, IP LPS injections for 4 weeks induces increased neutrophils in the periphery and brain, specifically the cortex, in mice. In our preliminary assessments, we found that neutrophil activation in the periphery peaked at 4 weeks of injections, therefore limiting our brain investigations to 4 weeks, but future studies should investigate the role of fewer and additional injections on neutrophils in the brain.

There are multiple mechanisms for how peripheral LPS may result in increased neutrophils in the brain. It has been demonstrated that LPS signaling through the TLR-4 receptor increases CD11b expression on neutrophils, and the binding of CD11b/CD18 (Mac 1) to ICAM one mediates adhesion and transmigration, which are necessary for extravasation into tissues^42^. We observed increased CD11b expression on peripheral and brain neutrophils following chronic low level LPS. Therefore, it is possible that LPS increases neutrophil adhesion and extravasation into the brain through increased CD11b expression. A previous study demonstrated that neutrophils enter the brain in 5xFAD mice, a mouse model for AD, through binding of LFA-1 to integrins, and LFA-1 dependent neutrophil recruitment has also been observed in a model of LPS-induced lung inflammation^14^. We did not measure LFA-1 (CD11a/CD18) on the surface of neutrophils in this study. However, this model can be used to further understand how peripheral inflammation may result in neutrophil migration into the brain and decipher which adhesion molecules are involved. In addition, peripheral inflammation can contribute to blood brain barrier (BBB) dysfunction, thus allowing for inflammatory cytokines and peripheral immune cells to more easily traffic into the brain and activate microglia that perpetuate neuroinflammation and continued BBB dysfunction^43^. Neutrophils have been found near areas of BBB dysfunction in AD mouse models but whether the BBB is a cause, or a consequence of neutrophil invasion remains to be determined^12^.

Increased CD11b mediates phagocytosis and oxidative burst in neutrophils and is increased on peripheral neutrophils in AD^37,44^, potentially due to increased TNFa^45–47^. In our study, TNFa increased 4 hours following the final LPS injection but resolved by 8 hours post-LPS and CD11b expression was sustained through 8 hours post-LPS in both the periphery and brain but resolved by 12 hours post-LPS. This suggests that weekly LPS injections do not result in sustained elevations of TNFa and neutrophil activation at this very low dose. It will be interesting in future studies to determine how sustained exposure to subclinical inflammatory stimuli may alter these responses in comparison to intermittent stimuli, which we tested here. However, previous studies have demonstrated that exposure to this level of LPS early in life resulted in cognitive impairments 10 months later in mice^48,49^, suggesting that damage caused by leukocytes that infiltrate temporarily may result in sustained impairment or may synergize later with age to contribute to degeneration. Finally, CD11b expression on neutrophils increases with age, so CD11b expression and neutrophil activation in mice receiving low-level chronic LPS should be further examined at different ages to determine how age impacts this model^50^.

Overall, the data provided here are evidence that chronic, low-level LPS administered in the periphery increases neutrophil frequency and activation in the periphery and brain. While we did not study functionality of neutrophils in this study, the observed increase in NE is suggestive of increased NET release, which has been observed as a peripheral marker of neurodegeneration and neuroinflammation^40^. NET release in the brain is known to contribute to damage, so future studies should investigate NETs in the brain following low levels of peripheral LPS exposure. Of note, neutrophils may also have suppressive functions and contribute to the resolution of inflammation^12^. Studies have demonstrated that LPS may induce regulatory T cells that in turn result in production of IL-10 by neutrophils, and this interaction is mediated by CD11b^51^. However, two observations suggest that is not a main mechanism observed in our study: 1) IL-10 peaks at 4 hours post-LPS while neutrophils remain elevated, suggesting that, at least at later timepoints, neutrophils are not producing high levels of IL-10; and 2) Neutrophils that produce IL-10 were demonstrated to have decreased CD11b expression in a prior study^51^, and neutrophils in this model upregulated CD11b expression following LPS. Future studies should perform functional assays to assess NET release, phagocytic ability, and ability to suppress T cell function to fully elucidate how neutrophils contribute to inflammation in this model.

This study has several limitations, some of which have already been discussed. First, this is a small pilot study and although we included both male and female mice, we are underpowered to analyze them separately. Sex differences should be examined in the future to understand how this model may be applied to investigate sexual dimorphism in neuroinflammation. Second, we observed some activation of peripheral neutrophils and some higher neutrophil counts in the brain with some of the saline-injected groups. This makes the interpretations of sustained neutrophil infiltration into the brain beyond 4 hours challenging. However, it depicts the continued need for control groups when performing injections and studying inflammation, as the injection itself may be a mediator of inflammation. Finally, we are unable to speak to the longevity of neutrophil responses with this model based on this pilot. Future studies should investigate sustained neutrophil inflammation beyond 12 hours following LPS injections and the potential for impacts weeks to months after injection. However, this study provides the foundation for an experimental model to induce neutrophil infiltration and activation in the brain with a subclinical peripheral stimulus, which can be used to understand the role of neutrophils in mediating the periphery-neuroinflammation axis in different mouse models of disease.

## Methods

### Animals

All mice in the study were generations F3 and F4 from the MRI in-house maintained strain C57BL/6J. The in-house lines are refreshed from The Jackson Laboratory periodically to reduce genetic drift. The mice were housed in individually ventilated and air filtered cages in a super-barrier mouse room. All cages, bedding, water, and enrichment were autoclaved, or UV treated prior to contact with the mice. Mice always had free access to food and water. All mouse cages were only opened in the hood and all personnel were wearing autoclaved lab coats and used sterile gloves. LPS and saline mice were co-housed to reduce cage to cage bias. In the 8-week study (Fig. 1A), 8 females and 11 males received LPS, and 7 females and 8 males received saline. In the subsequent 4-week study (Fig. 1C), LPS injected mice had 6 females and 6 males received LPS 6 females and 5 males received saline.

### LPS injections

Mice were weighed weekly for 0.5 mg/kg by weight calculations. Vaccine grade LPS from *Escherichia coli* 0111:B4 (InvivoGen) or United States Pharmacopeia (USP) sterile grade saline (Cytiva Z1376), were 0.22 uM sterile filtered prior to injections. Mice were injected IP with LPS or sterile saline once a week for 8 weeks for the preliminary study (Fig. 1A), and once a week for 4 weeks for the next iteration (Fig. 1C).

### Blood and brain collection

Baseline blood was collected the day before the first injection. In the initial experiment, submandibular blood was collected 12 hours after the 4-week and 8-week (final) LPS or saline injection. For the next iteration of the study, submandibular blood was collected at 12, 8, or 4 hours after the 4-week (final) LPS or saline injection just prior to euthanasia and brain collection. Following the submandibular bleeds, deep anesthetization was carried out with avertin via IP injection. A cardiac puncture was then performed to obtain additional blood prior to whole body perfusion with 20 mL of PBS to flush the vasculature (until fluids ran clear). Following perfusion, the brain was then removed and placed in R10 (10% fetal bovine serum in RPMI 1640 with L-glutamate and 25 mM HEPES) and kept on ice until tissue processing.

### Brain tissue processing

One hemibrain was enzymatically digested with media (RPMI 1640 with L-glutamate and 25 mM HEPES) supplemented with Liberase (40 μg/ mL, Sigma-Aldrich) and DNAse (4μg/mL, Sigma-Aldrich) for 45 minutes at 37 C with vigorous stirring and ground through a 70-μm cell strainer, as previously described (PMID: 29907866). Isolated brain leukocytes were separated via a percoll gradient as previously described (PMID: 34036283) for flow cytometry analysis. The second hemibrain was fixed in 10% buffered formalin for 24 hours and then transferred to 70% ethanol until the paraffin-embedding procedure for microscopy staining.

### Flow cytometry

All blood and processed brain hemi samples were stained using anti-mouse recombinant antibodies: Ly6G BV421 (Biolegend, Clone 1A8), CD45 APC (Biolegend, Clone 30-F11), CD62L PE (Biolegend, Clone MEL-14), and CD11b (Biolegend, Clone M1/70) along with a fixable live/dead stain (Invitrogen L34988). Stained samples were fixed with a 1x fixation buffer (Biolegend 420801) and CountBright^™^ beads (Invitrogen C36950) were added prior to data acquisition. Fluorescence data was collected by flow cytometry on a Sony SH800S cell sorter. FlowJo (version 10.9.0, BD Biosciences) was used to analyze the data. Cells were gated based on antibody markers and/or scatter. Absolute neutrophil frequencies were calculated for peripheral blood based on the percentage of CountBright^™^ beads collected.

### Soluble analyte analyses

Cytokine and chemokines involved in neutrophil mobilization and activation were measured using a custom multiplex bead array kit (LEGENDPlex, Biolegend). Twelve cytokines were measured including, CXCL1, CXCL2, G-CSF, IL-1b, IL-6, IL-4, TGFb, TNFa, GM-CSF, INFg, IL-17A, and IL-10. The assay was performed according to manufacturer’s instructions in duplicate, collected on an SH800 (Sony), and analyzed using the LEGENDPlex Cloud-Based Data Analysis Software (Biolegend and Qognit). Neutrophil elastase was measured via ELISA using a pre-validated kit following manufacturer’s instructions (Abcam) and read using a Mini ELISA Plate Reader (Biolegend).

### Immunohistochemistry

Formalin-fixed paraffin embedded sections (5 μm thickness) were baked at 60°C for 20 minutes, dewaxed in xylene for 1 hour, and rehydrated through an alcohol series. Antigen retrieval with 1X Reveal Decloaker (Biocare Medical) was performed using the capillary gapping method with a vegetable steamer for 35 minutes. Once sections were cooled and washed, they were blocked in Background Sniper (Biocare Medical) for 10 minutes. Primary antibodies, Chicken anti-Mouse/Human/Rat GFAP (Invitrogen PA1–10004) and Goat anti-Human/Mouse MPO (R&D AF3667) were diluted to 1 μl/mL and 0.5 μl/mL, respectively, in sniper and added to sections overnight at 4°C. Primaries were removed, and sections were washed in TBST twice before species-specific secondary antibodies Donkey anti-Chicken Ax594 at a 2 μl/mL concentration and Donkey anti-Goat IgG Ax488 at a 4 μl/mL concentration were added for 2 hours at room temperature. A TBS with 0.1% Triton X wash and distilled water wash performed, and then coverslips were mounted onto sections with Vectashield HardSet Antifade Mounting Medium with DAPI (Vector Laboratories) before imagining. Sections were imaged at 20x or 40x on an Olympus Fluoview FV1000 and neutrophils were counted on a Zeiss Axio Imager.M1 (20x and 40x objective).

## Data Availability

The datasets generated and analyzed during this study are available from the corresponding author upon reasonable request.
